# Time-based task expectancy: perceptual task indicator expectancy or expectancy of post-perceptual task components?

**DOI:** 10.1007/s00426-021-01588-1

**Published:** 2021-11-16

**Authors:** Irina Monno, Stefanie Aufschnaiter, Sonja Ehret, Andrea Kiesel, Edita Poljac, Roland Thomaschke

**Affiliations:** Cognition, Action and Sustainability Unit, Department of Psychology, Albert-Ludwigs-Universität Freiburg, Engelbergerstrasse 41, 79085 Freiburg, Germany

**Keywords:** Task switching, Time-based expectancy, Temporal preparation, Foreperiod

## Abstract

The temporal predictability of upcoming events plays a crucial role in the adjustment of anticipatory cognitive control in multitasking. Previous research has demonstrated that task switching performance improved if tasks were validly predictable by a pre-target interval. Hence, far, the underlying cognitive processes of time-based task expectancy in task switching have not been clearly defined. The present study investigated whether the effect of time-based expectancy is due to expectancy of post-perceptual task components or rather due to facilitation of perceptual visual processing of the coloured task indicator. Participants performed two numeric judgment tasks (parity vs. magnitude), which were each indicated by two different colours. Each task was either more or less frequently preceded by one of two intervals (500 ms or 1500 ms). Tasks were indicated either by colours that were each more frequently (or in Exp. 1 also less frequently) paired with the interval or by colours that were equally frequent for each interval. Participants only responded faster when colour and task were predictable by time (expected colour), not when the task alone was predictable (neutral colour). Hence, our results speak in favour of perceptual time-based task indicator expectancy being the underlying cognitive mechanism of time-based expectancy in the task switching paradigm.

## Introduction

In cognitive multitasking research, it is a central and well-established finding that participants respond faster and with fewer errors when the task is repeated rather than being switched in consecutive trials. The detrimental performance effects of switching between tasks are referred to as switch costs (Monsell, 2003). One of the key findings within this research field is that performance improves when participants are informed in advance about a specific task requirement.

This performance improvement can be attained if tasks are able to be predicted by prior events (see Broeker et al., 2017 for review on predictability). Task predictability is induced in several ways, such as through the predictability of task sequences (Gotler et al., 2003; Heuer et al., 2001; Koch, 2001) or the predictability of a proportion of task switches (Bonnin et al., 2011; De Baene & Brass, 2013; Dreisbach & Haider, 2006; Duthoo et al., 2012). For instance, when participants are initially trained to execute two different tasks in a fixed sequence, their performance improves when the tasks are presented in the previously learned sequence and are therefore predictable, when compared with the performance when the task sequence is random (Koch, 2001, 2005).

Task switching studies typically use explicit cues to inform the participants about the upcoming task (for an overview see Kiesel et al., 2010). Performance often benefits from early task cue presentations (Koch, 2003). Furthermore, extending the time interval between task cue and target stimulus also seems to be beneficial to performance (Arrington & Logan, 2004b; Koch, 2001; Logan & Bundesen, 2003; Logan & Schneider, 2006; Rogers & Monsell, 1995). It was concluded that the preparation processes that take place between cue and stimulus presentation are advantageous, if sufficient time is available for task preparation.

More recently, it has been suggested that the duration of the time interval itself can serve as a cue for an upcoming event, thus enabling preparation for fulfilment of the task. By frequently instructing specific tasks after specific foreperiods, Aufschnaiter and colleagues (2018a, b) demonstrated that time-based expectancy in task switching contexts improved multitasking performance.

## Time-based expectancy

Effects of temporal predictability have an impact on many basic aspects of cognition, such as attention, perception, learning and memory (Nobre & van Ede, 2018). For instance, when time correlates with an event, humans form time-based expectancy. Such time-based expectancy is typically investigated by the time-event correlation paradigm (Thomaschke & Dreisbach, 2015; Wagener & Hoffmann, 2010). In this paradigm, time-based expectancy is formed by increasing the frequency of combinations used between foreperiods and specific events, leading to a more frequent occurrence of particular events after a certain time interval than for other events. For instance, Wagener and Hoffmann (2010) combined two target stimuli with two different foreperiods (600 and 1400 ms). The appearance of one stimulus was four times more likely after the short foreperiod than after the long foreperiod and the reverse was true for the other stimulus used. The authors observed improved performance (faster responses and fewer errors) when using frequent foreperiod-stimulus combinations as compared to less frequent foreperiod-stimulus combinations. According to time-based event expectancy theory (Thomaschke & Dreisbach, 2015), connections between the successive mental representation of points in time and expectancy of specific events become conditioned during learning with the time-event correlation paradigm. Thus, after some practice, a represented point in time associated with the time lapse automatically activates expectancy of the event which is most likely to occur.

Note that time-based expectancy is conceptually very different from the more widely researched phenomenon of temporal expectancy. Temporal expectancy (or temporal preparation) refers to the expectancy of a certain foreperiod, that is, for the occurrence of any target stimulus at a certain point of time. Temporal expectancy can be induced by the distribution of foreperiods in an experiment (e.g. more short than long foreperiods (Zahn & Rosenthal, 1966) or by cues before the foreperiod, which signal its duration (Coull et al., 2000; Kingstone, 1992). In time-based expectancy, on the contrary, a certain foreperiod is not expected but rather a certain type of target conditional upon a foreperiod. For example, target A may be expected after a short foreperiod and target B after a long foreperiod, given that both foreperiods occur equally frequent. Time-based expectancy is typically induced by correlations between a foreperiod and targets (Thomaschke & Dreisbach, 2013; Volberg & Thomaschke, 2017; Wagener & Hoffmann, 2010). Moreover, time-based expectancy has already been demonstrated in other domains such as visual stimulus perception (Thomaschke et al., 2016), attentional adjustment to conflict contingencies (Wendt, & Kiesel, 2011), language processing (Roberts & Francis, 2013; Roberts et al., 2011; Roberts & Norris, 2016), human-machine interaction (Shahar et al., 2012; Thomaschke & Haering, 2014) or the perception of emotional word valence (Thomaschke et al., 2018). These studies typically examined time-based expectancy in single tasks.

Multitasking performance can also profit from temporal predictability of events. Aufschnaiter et al. (2018a, 2018b) combined the standard cuing task switching paradigm and the time-event correlation paradigm (Thomaschke & Dreisbach, 2013; Thomaschke et al., 2015; Wagener & Hoffmann, 2010). Participants categorized number stimuli either according to parity or magnitude (smaller/larger than 5). The colour of the presented number indicated which of the two tasks had to be performed in the trial. The two tasks were combined with two different foreperiods (short and long), such that one task was more likely after the short foreperiod, whereas the other task was more likely after the long foreperiod. The degree of predictability varied across the experiments (90% in Experiment 1, 80% in Experiment 2 and 70% in Experiment 3). Participants were faster and usually more accurate in trials with predictable combinations of foreperiod and task type. These findings suggest that the participants formed strong associations between frequent foreperiod-task combinations and were able to use this temporal information to prepare for processing the associated task set. According to Logan and Gordon (2001), the task set entails the certain task parameters that define stimulus categorization and response mapping rules that are more or less activated in working memory. Thus, time-based task expectancy seems to facilitate the implementation of those rules for the specific task.

However, the results observed in the study of Aufschnaiter et al. (2018a, 2018b) might also be explained differently. Instead of assuming the retrieval of a task set due to temporal information, preparation of perceptual processes might also have taken place. In the study of Aufschnaiter et al. (2018a, 2018b), task indicators were presented as a part of the stimulus feature (i.e. its colour). Thus, the foreperiod was not only correlated with the task but also with the colour. Recent studies have shown that temporal preparation additionally speeded up perceptual processes (Rolke, 2008; Rolke & Ulrich, 2010; Seibold & Rolke, 2014a, 2014b; Steinborn et al., 2008, 2010). There is also empirical evidence for time-based expectancy due to visual stimuli (Rieth & Huber, 2013; Thomaschke et al., 2016). Rieth and Huber (2013) observed that spatial attention implicitly adapts to contingencies involving combinations of time and space and that learning these combinations influences the future performance in trials without these spatial-temporal contingencies. In addition, Thomaschke et al. (2016) examined whether time-based expectancy assists the processing of visual stimulus features or response processing in an environment, where temporally predictable stimulus features are crucial for the choice of reaction. A strong time-based expectancy effect was only observed in trials where a stimulus feature and response were simultaneously predicted by associating them with the foreperiod, showing that time-based expectancy can affect perceptual processing (Thomaschke et al., 2016). Similarly, the performance improvement observed by Aufschnaiter et al. (2018a, 2018b) can reflect both the time-based expectancy for the task indicator (i.e. colour) and/or the time-based expectancy for the task set itself.

This conclusion finds support in the study by Schröter et al. (2015). The authors manipulated task expectancy throughout blocks of trials. In blocks with expected tasks, participants constantly switched between two tasks and thus always knew which task to perform in the upcoming trial. In blocks with unexpected tasks, they constantly switched between three tasks in an unpredictable manner. A larger variable foreperiod effect, i.e. faster responses to the long foreperiod compared to the short foreperiod, was observed when one of the tasks was expected than when it was not, providing evidence for the task-specific temporal preparation. Thus, the findings of Schröter et al. (2015) support the hypothesis that time-based expectancy may facilitate task processing. Given the evidence that temporal preparation can affect both the visual stimuli processing (Seibold & Rolke, 2014a, 2014b) and task set processing (Schröter et al., 2015), in the present study, it was aimed to extricate the time-based expectancy for the task indicator (i.e. colour) from the time-based expectancy for the task set. Therefore, two experiments were conducted, in which four colour task indicators for two tasks were used. In Experiment 1, the task switching performance was examined in two experimental conditions, which exactly replicated the setting used in the Aufschnaiter et al. (2018a, 2018b) studies. Additionally, in Experiment 1 and 2 task switching performance was also examined in a setting in which the specific foreperiod correlated exclusively with the specific task.

More precisely, in the present study, the experimental design was extended to include four colours as tasks indicators instead of only the two colours used by Aufschnaiter et al. (2018a, 2018b). Two of the colours indicated the magnitude judgment task, while the other two colours indicated the parity judgment task. This enabled different experimental settings to be created, where correlations with time were selective (see Table 1 for the overview of the trial frequencies for each block in Experiment 1 and Experiment 2). Specifically, the frequencies of foreperiod-colour combinations are arranged in a way that in each trial, given the current foreperiod, four different types of trials can occur: (i) A colour and a task, which are both expected (i.e. are likely, because they appear more often after the current foreperiod than after the other foreperiod); (ii) A colour and a task, which both are unexpected (i.e. are unlikely, because they appear more often after the other foreperiod than after the current foreperiod), (iii) A colour that is neutral (i.e. neither particularly likely nor particularly unlikely, because it occurs as often after the current foreperiod as after the other one), with a task that is expected (i.e. is likely, because it occurs more often after the current foreperiod than after the other foreperiod), (iv) A colour that is neutral (i.e. neither particularly likely nor particularly unlikely, because it occurs as often after the current foreperiod as after the other one), with a task that is unexpected (i.e. is unlikely, because it occurs more often after the other foreperiod than after the current foreperiod). Trials of the second case (ii) could only occur in Experiment 1. In total, our design allows for distinguishing between three different variations of events: *expected*, *unexpected* and *neutral*.

**Table 1:** Trial frequencies per block

Foreperiod (ms)	Experiment 1				Experiment 2			
	Task and colour expectancy		Task expectancy		Task and colour expectancy		Task expectancy	
	T1 + C1	T2 + C2	T1 + C3	T2 + C4	T1 + C1	T2 + C2	T1 + C3	T2 + C4
500	36	4	20	20	40	0	20	20
1500	4	36	20	20	0	40	20	20

*T* task, *C* colour

It might be argued that the present arrangement favours a selective perceptual preparation, as a specific foreperiod correlates with a specific colour more frequently than with a specific task (see Table 1). On the other hand, there are two opportunities to detect the time-based task expectancy in the task switching scenario, either as a unique effect or as an additive impact of temporal preparation for visual stimuli processing and for a task set. Therefore, to examine whether evidence is found for different forms of temporal preparation, we decided on the following analyses: Firstly, it was assessed whether there is time-based expectancy for colour and task. For this, frequent vs. infrequent combinations of a foreperiod with colour and task were compared in Experiment 1 (see Expected Colour and Task vs. Unexpected Colour and Task). Given that there was no infrequent combination of foreperiod with colour and task in Experiment 2, expected colour and task were compared with neutral colour and unexpected task. Secondly, to assess whether time-based expectancy remains even when colour is equally frequent, i.e. whether there is time-based expectancy for a task, neutral colour and expected task were compared with neutral colour and unexpected task. Finally, to disentangle between time-based expectancy for colour and time-based expectancy for a task, expected colour and task were compared with neutral colour and expected task. The absence of a difference in performance would speak in favour of time-based expectancy for task exclusively. The performance advantage in expected colour and task would indicate time-based expectancy for both colour and task. In other words, time-based expectancy would accelerate visual processing of the task indicator and, in addition, facilitate the task set processing.

Furthermore, in task switching scenarios, cognitive processes in task switch and task repetition trials differ. Task switches require implementation of a new task set and involve processes such as inhibition of the previous task and activation of the current task. In task repetitions, only the previous task-set needs to be kept active. This results in so-called switch costs, meaning slower and more error-prone responses when switching than when repeating tasks (see Kiesel et al., 2010 and Koch et al., 2018 for reviews). If time-based expectancy facilitates any of those processes only involved in switch trials, an interaction should be expected between time-based expectancy and the factor transition (i.e. task repetition compared to task switches). Such an interaction was only observed in Experiment 1 in the study of Aufschnaiter et al. (2018b), whereas in most experiments task transition did not interact with time-based expectancy (see Aufschnaiter et al., 2018a, 2018b, except Experiment 1; 2020; 2021). We still included the factor transition as well the additional factor foreperiod for each of the analyses in order to elaborate potential interactions.

In total, the present approach enables the disentangling of the time-based expectancy for perceptual components from the time-based task set expectancy and also examines the potential impact of the two types of time-based expectancy on performance in a task switching scenario.

## Experiment 1

In a previous study (Aufschnaiter et al., 2018a), robust time-based expectancy effects were observed in predictable environments with 90%, 80% and even 70% validity between foreperiod and colour and task. In Experiment 1, it was decided on a design in which time-based expectancy for task was induced with 70% validity. At the same time, time-based expectancy for the task indicator was induced with 90% validity. With the time-based expectancy effect resulting in faster response times (RTs) and lower error rates (ERs), we assumed faster and fewer erroneous responses for expected than for unexpected or neutral events.

## Method

### Participants

64 subjects (50 females) participated in the experiment. They were students from the University of Freiburg or residents of Freiburg who received either course credits or financial remuneration for their participation. All participants had normal or corrected to normal vision, 53 of them were right-handed. The mean age was 23.75, *SD* = 5.16. One participant was excluded due to technical problems. The sample size of 64 participants was calculated for detection of a medium-sized effect of time-based expectancy. The effect size η_p_^2^ =  .24 from Experiment 1 (90% predictability)^1^ was used in the study of Aufschnaiter et al. (2018a). A priori power analysis (*α* = .05, 1 − β =  .8) yielded a minimum number of 46 participants. For counterbalancing reasons, the number of participants was increased to *N* = 64.

### Apparatus and stimuli

The experiment was run in a dimly lit room. Participants were placed in front of a computer screen at a viewing distance of approximately 50 cm. They responded by pressing the keys “y” or “m” with their left or right index finger on a standard QWERTZ keyboard, which was positioned centrally on the table. All stimuli were presented in the centre of the screen. Target stimuli were coloured (yellow, green, red or blue) numbers from 1 to 9, except 5, measuring approximately 8 × 5 mm, presented against a black background. The warning stimulus was a white “ + ” symbol (Arial typeface), measuring 6 × 6 mm.

### Procedure

**Fig. 1:**
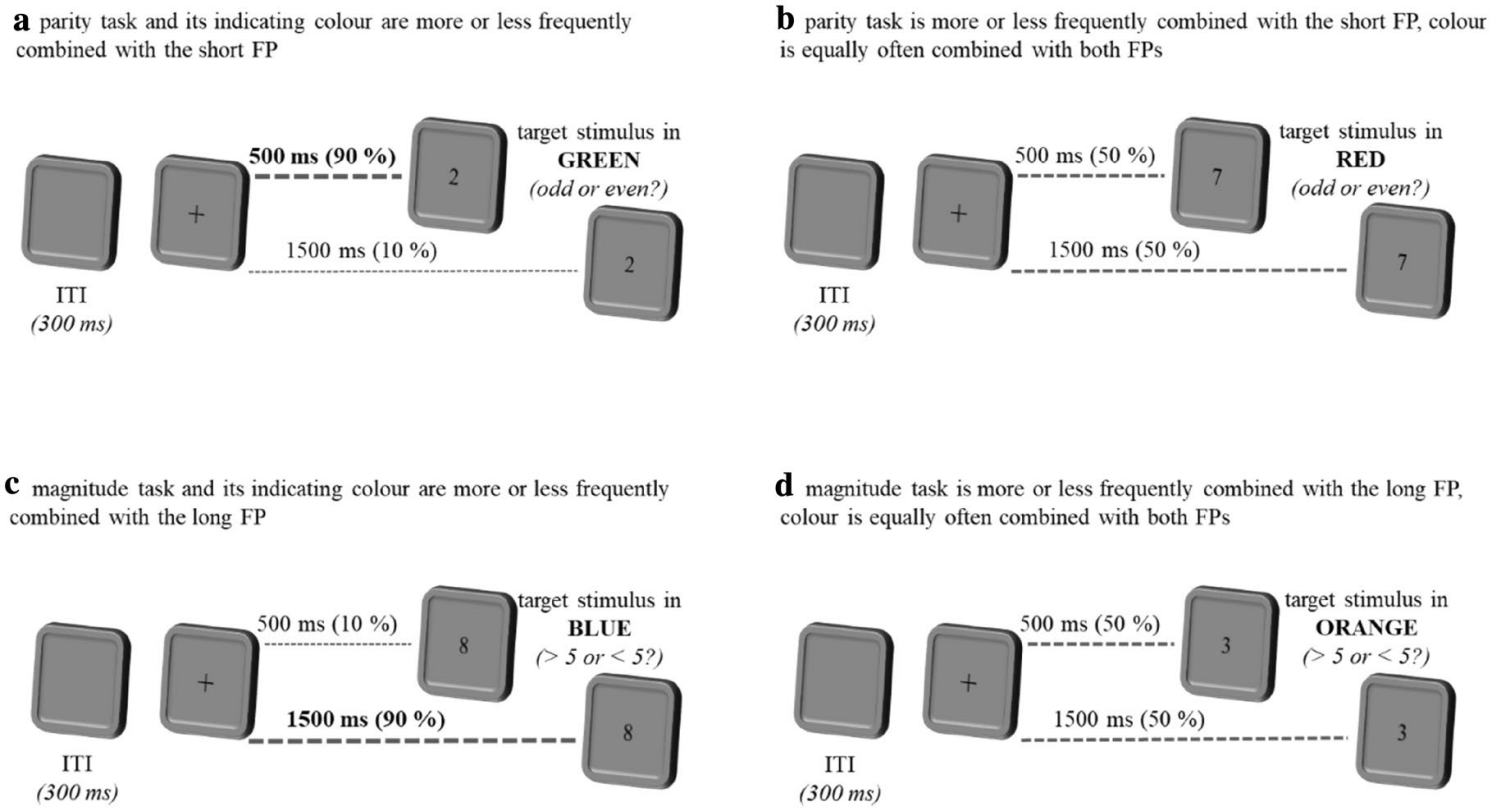
Example of the trial procedure in Experiment1 with different combinations of foreperiod, colour and task, resulting in 90% colour predictability and 70% task predictability. In **a** and **c** the specific task and its indicating colour are both either expected or unexpected. In **b** and **d** the other two task-indicating colours are neutral (50% of occurrence after both FPs). But the tasks are either still expected or unexpected

**Fig. 2:**
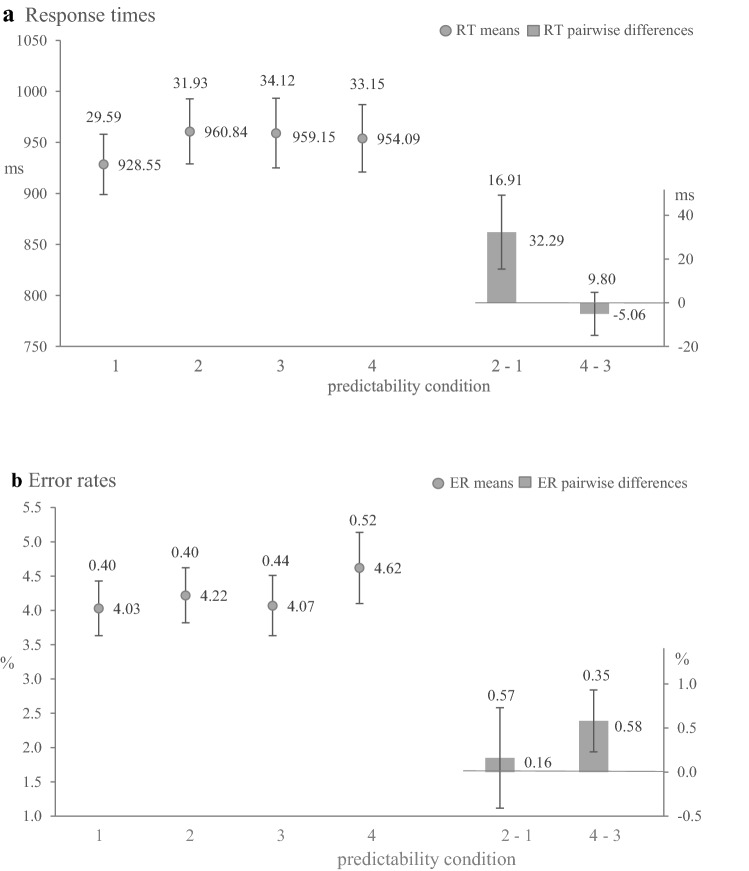
Results of analysis in Experiment 1.** a** Mean RTs for each condition (dots; 1 = expected colour and task, 2 = unexpected colour and task, 3 = neutral colour and expected task, 4 = neutral colour and unexpected task) and corresponding pairwise differences (bars).** b** Mean ERs for each condition (dots) and corresponding pairwise differences (bars). Numbers beside the dots and the bars indicate the numerical values of means. Error bars represent ± 1 SEM, numbers above error bars are the numerical values of the SEMs. * *p* < .05. The results are averaged across FPs and task transition

At the beginning of each trial, a blank screen was presented for 300 ms (inter-trial interval, ITI). Then a fixation cross appeared, representing the warning interval (foreperiod). The foreperiod was either 500 ms or 1500 ms long. After the foreperiod, the target stimulus was presented until a response was elicited for the participant (for an illustration of the design and exemplary trial procedure, see Fig. 1). The colour of the target stimulus indicated whether the participants had to perform a parity or a magnitude judgement task in the current trial. Thus, the colours served as explicit task indicators. Two colours indicated the magnitude task to be performed, while the other two colours indicated the parity task to be performed. Two of the colours, one indicating the magnitude task and one indicating the parity task, were displayed at different frequencies after both foreperiods. One of these colours followed the short foreperiod in 90% of its occurrences, while the other colour followed the long foreperiod in 90% of its occurrences (Fig. 1a, c). The two other colours, one similarly indicating the magnitude task and one indicating the parity task, were displayed equally frequently after both foreperiods. This means that each of these colours appeared equally often after the short and after the long foreperiod (Fig. 1b, d). In this way, two of the colours were temporally predictable while the other two were not. Yet the task was also temporally predictable with a 70% probability. As shown in Table 1 (see column “Experiment 1”), this scenario resulted in four different types of trials: expected colour and task, unexpected colour and task, neutral colour and expected task, as well as neutral colour and unexpected task (Fig. 2).

The order of the trials was random, but the magnitude and the parity judgment tasks were presented equally often. The assignment of tasks to foreperiods was counterbalanced across participants, whereas the mapping of colours to tasks was only partly counterbalanced between different conditions. Specifically, for half of the participants, the colours “blue” and “orange” indicated the magnitude task and the colours “green” and “red” the parity task. The colours “blue” and “green” were thus correlated either with a short or a long foreperiod, but the colours “red” and “orange” occurred equally often after both foreperiods. For the other half of the participants, the colours “green” and “red” indicated the magnitude task and the colours “blue” and “orange” the parity task. Now the colours “red” and “orange” were correlated either with the short or the long foreperiod, while the colours “green” and “blue” were not. The participants were not informed that the foreperiods were of different duration, nor that the duration correlated with any of the upcoming tasks. Participants were instructed to respond as fast and as accurate as possible. When they committed an error, the white-coloured word *Fehler!* (German for “Error!”) appeared on a black screen for 1500 ms.

The experiment included four blocks of 160 trials each. In the first (learning) block, an abbreviated version of the instruction appeared for 8000 ms on a black screen after a wrong response. The three following blocks were test blocks without any instructions after errors. After each block, participants could take a self-paced break. A day before the main session, participants completed a training session, which was the same as the main experiment. One session lasted 45 min. After the main session of the experiment, participants were asked whether they had noticed any temporal regularities.

## Analysis

To examine our three hypotheses, we planned to run three separate 2 × 2 × 2 repeated measures ANOVAs, with the factors foreperiod (500 vs. 1500 ms), transition (repetition vs. switch) and temporal predictability condition which included different types of trials, for RTs and ERs. We assumed that time-based task expectancy effect would be reflected in performance benefits (i.e. lower RTs and ERs) in all task expected trials, i.e. with expected and neutral colours. Thus, in the first analysis we examined whether the RTs and ERs in trials with expected colour and task (the first condition) are significantly smaller than the RTs and ERs in trials with unexpected colour and task (the second condition). In the second analysis, RTs and ERs were compared in trials with a neutral colour and expected task (the third condition) against RTs and ERs in trials with a neutral colour and unexpected task (the fourth condition). The significant lower RTs and ERs for expected events in the first and the second analyses would indicate time-based expectancy effect on task and potentially also on colour. If this were the case, further analysis was planned to differentiate between time-based expectancy for colour and time-based expectancy for task by contrasting trials with expected colour and task (the first condition) against trials with neutral colour and expected task (the third condition). The significantly lower RTs and ERs in the first condition than in the third condition would speak in favour of the additive effect of time-based colour and task expectancies, whereas the absence of difference would confirm that time-based expectancy effect is exclusively task-related. Please note that this third analysis would not be conducted if the significantly lower RTs and ERs were observed only in trials with expected colour and task (first ANOVA) but not in trials with neutral colour and expected task (second ANOVA), because that would provide clear evidence in favour of time-based colour expectancy.

In addition, for each separate ANOVA a corresponding Bayesian repeated measures ANOVA was run with default prior scales using the open-source statistical software program, JASP (JASP Team, 2016). The Bayesian ANOVA compared the different models to the null model and provided information about their relative adequacy. Our main goal was to evaluate the evidence for the absence of a difference between the temporal predictability conditions, thus the Bayes Factor was examined for the null model (*BF*_*01*_). According to the interpretation from Lee and Wagenmackers (2013; adjusted by Jeffrey, 1961) *BF* lower than 1 represents “no evidence”, *BF* between 1 and 3 “anecdotal evidence”, *BF* between 3 and 10 “moderate evidence, *BF* from 10 to 30 “strong evidence”, *BF* from 30 to 100 “very strong evidence”, and *BF* larger than 100 “extreme evidence”. On the basis of the results of the frequentist analysis, the other significant main effects (foreperiod and/ or transition) were included in the null model and compared this model against the alternative model which entailed the main effect of predictability.

## Results

As in previous studies on time-based expectancy (Aufschnaiter et al., 2018a; Thomaschke, & Dreisbach, 2013), we analysed only data from the second session. Before conducting the analyses, we excluded the first learning block, the first three trials of each block, trials with RTs < 100 ms, trials with number repetition, as well as trials following an error. Erroneous trials were also excluded from the RT analyses. Before the RT analyses, we further removed the trials with RTs more than three SDs away from their condition mean for each combination of block, frequency, foreperiod, type of transition and participant. The screening procedure described above followed exactly the procedures employed in previous studies in the area of time-based expectancy in task switching (Aufschnaiter et al., 2018a). In the following reports, we focused on the significant results. Tables 2 and 3 in the appendix provide an overview of all the results of ANOVAs.

### Expected colour and task vs. unexpected colour and task

**Response Times** The main effect of foreperiod, *F* (1, 62) = 14.32, *p* =  .00, η_p_^2^ = .19 and the main effect of transition, *F* (1, 62) = 42.58, *p* = .00, η_p_^2^ = .41, were significant. Furthermore, participants showed the tendency to faster responses if the task and the colour were expected than if both the task and the colour were unexpected, but the main effect of temporal predictability did not reach significance, *F* (1, 62) = 3.65, *p* =  .06, η_p_^2^ =  .06 (pairwise differences 2 - 1, Fig. 1a). There were no significant interactions between the factors. To evaluate the evidence for the lack of difference between the two predictability conditions, we additionally performed a Bayesian repeated measures ANOVA. We included the main factors of foreperiod and transition in the null model and compared this model against the alternative model, which comprised the main effect of predictability, as well as against the alternative models that also included the interactions. The Bayes factor indicated that the data were about 2.4 times more likely under the null model than under the alternative model which included the factor predictability condition, *BF*_*01*_ = 2.39. That means that there is only anecdotal evidence (Lee & Wagenmakers, 2013) for the absence of a time-based expectancy effect. With regard to interactions, the corresponding Bayes factors provided moderate to strong evidence for the null model without interactions, *BF*_*s01*_ > 7.62.

**Error Rates** Participants produced significantly fewer errors in trials with tasks repetitions compared to trials with tasks switches, *F* (1, 62) = 4.52, *p* = .04, η_p_^2^ = .07. Neither of the main effects, nor any of the interactions were significant. An analogous Bayesian repeated measures ANOVA showed that the observed data were almost 10 times more likely under the null model than under an alternative model which comprised the main effect of predictability condition, *BF*_*01*_ = 9.51, as well as about 8 times more likely than under the alternative model which comprised the main effect of foreperiod, *BF*_*01*_ = 8.67.

### Neutral colour and expected task vs. neutral colour and unexpected task

**Response Times** Two significant main effects of foreperiod *F* (1, 62) = 17.35, *p* = 0.00, η_p_^2^ = .22 and of transition *F* (1, 62) = 36.03, *p* = .00, η_p_^2^ = .37 were observed, but there was no main effect of temporal predictability condition *F* (1, 62) = .26, *p* = .61, η_p_^2^ = .00 (pairwise differences 4 - 3, Fig. 1a). There were no significant interactions between the factors. An analogous Bayesian repeated measures ANOVA provided strong evidence for the absence of a time-based expectancy effect. The factors foreperiod and transition, were again included in the null model. The Bayes factor indicated that the observed data were about 9 times more likely under the null model than under the alternative model which comprised the main effect of predictability condition *BF*_*01*_ = 9.12. There were no significant interactions between the factors.

**Error Rates** We observed a significant main effect of task transition, *F* (1, 62) = 4.79, *p* = .03, η_p_^2^ = .07, as well as a significant interaction of foreperiod and transition, *F* (1, 62) = 5.82, *p* = .02, η_p_^2^ = .09. The main effect of predictability condition gained no significance, *F* (1, 62) = 2.74, *p* = .1, η_p_^2^ = .04. The Bayesian repeated measures ANOVA revealed moderate evidence for the absence of any time-based expectancy effect, *BF*_01_ = 3.60 and strong evidence for the absence of any main effect of the foreperiod, *BF*_*01*_ = 1.

**Post-Experimental Questionnaire.** According to the post-experimental interview, none of the participants noticed any temporal regularity in the experiment.

## Discussion

In Experiment 1, we investigated whether the time-based expectancy effect encountered in task switching (Aufschnaiter et al., 2018a, 2018b) was related either to the perceptual processing of the task indicator or to the retrieval of the task-set or an accumulation of both processes. The RTs showed a tendency to faster responses in trials with expected colour and task compared to trials with unexpected colour and task. There was no difference between trials with neutral colour and expected task and trials with neutral colour and unexpected task. Although the main effect of the predictability condition was not significant, the corresponding Bayesian analysis could not provide convincing evidence for or against the time-based colour expectancy, whereas there was a strong evidence against time-based task expectancy. The analyses of the ERs did not yield any traces of the time-based expectancy effect. Based on the present findings, it was refrained from performing the third analysis.

In accordance with previous findings, we observed faster responses for short foreperiods and for task repetitions (Aufschnaiter et al., 2018a, 2018b) and found no evidence for time-based expectancy in ERs (Aufschnaiter et al., 2018a, 2018b; Thomaschke & Dreisbach, 2015).

Generally speaking, the results obtained here provided only a tentative hint that the previously observed (Aufschnaiter et al., 2018a, 2018b) time-based expectancy effect in task switching is more due to colour expectancy than to task expectancy. Therefore, we conducted Experiment 2 to further investigate the observed trend.

## Experiment 2

Although Aufschnaiter et al. (2018a) found time-based expectancy effects on performance in task switching, even with a 70% probability of predicting the upcoming event from the foreperiod (colour and task), we did not observe time-based expectancy in a comparable combination of foreperiod with colour and task in Experiment 1. However, the present experimental design included four colours as task indicators instead of the two used in previous studies (Aufschnaiter et al., 2018a, 2018b). Assuming that learning four different colour-task combinations is cognitively more challenging and might interfere with the learning of foreperiod-event associations, we intended to strengthen the induction of time-based expectancy effect in Experiment 2. Thus, a predictability condition was created, where the foreperiod led to the prediction of the upcoming task with 75% validity and the task indicator with 100% validity.

## Method

### Participants

64 subjects (49 females) participated in the experiment. They were students from the University of Freiburg or residents of Freiburg and received course credit or remuneration for their participation. All participants had normal or corrected to normal vision, 57 of them were right-handed. The mean age was 23.48, *SD* = 3.9.

### Procedure

Apparatus and stimuli were identical to Experiment 1. The procedure was also in accordance with Experiment 1 except for the strengthened induction of expectancy. Now two of the colours, one indicating the magnitude task to be performed and one indicating the parity task to be performed, followed a specific foreperiod in 100% of its occurrences. The two remaining neutral colours, also each indicating the respective tasks, were equally frequently displayed after both foreperiods. This meant that each of these two colours appeared equally often after the short and after the long foreperiod. Consequently, now the task occurred after the specific foreperiod with a 75% probability.

As a consequence, there were in total three types of trials: expected colour and task, neutral colour and expected task, neutral colour and unexpected task (see column “Experiment 2” in Table 1). Therefore, the temporal predictability conditions in Experiment 2 were equal to the predictability conditions 1, 3 and 4 used in Experiment 1.

### Analyses

As in Experiment 1, we planned to run three separate 2 × 2 × 2 repeated measures ANOVAs, with the factors foreperiod (500 vs. 1500 ms), transition (repetition vs. switch) and predictability condition which included different types of trials, for RTs and ERs separately. Due to the strengthened induction of time-based expectancy, there were no trials with unexpected colour and task (Table 1, column “Experiment 2”). Therefore, in the first ANOVA, we compared the RTs and ERs in trials with expected colour and task (first condition) against RTs and ERs in trials with neutral colour and unexpected task (fourth condition). In the second analysis, we compared RTs and ERs in trials with neutral colour and expected task (third condition) and in trials with neutral colour and unexpected task (fourth condition). If time-based expectancy effect had been observed in the first and in the second analyses, a third analysis would have been planned to examine the difference between performances in trials with expected colour and task (first condition) and in trials with neutral colour and expected task (third condition). The significantly lower RTs and/or ERs in the first condition than in the third condition would indicate time-based expectancies for colour and for task, whereas the absence of difference would confirm that the time-based expectancy was exclusively task related.

In addition, to examine whether the non-significant time-based expectancy effect in Experiment 1 was due to its weakness in inducing time-based expectancy, we conducted a cross-experiment analysis. A mixed-design ANOVA contained within-subjects factors foreperiod (short vs. long), transition (repetition vs. switch), predictability (expected colour and task vs. neutral colour and unexpected task) and the between-subjects factor – the experiments (1 vs. 2). We used trials with neutral colour and unexpected task in cross-experiment analysis due to the lack of trials with unexpected colour and task in Experiment 2. We also ran a corresponding Bayesian analysis for each separate ANOVA and included the other main effects (foreperiod and/or transition) in the null model if they were significant.

## Results

**Fig. 3:**
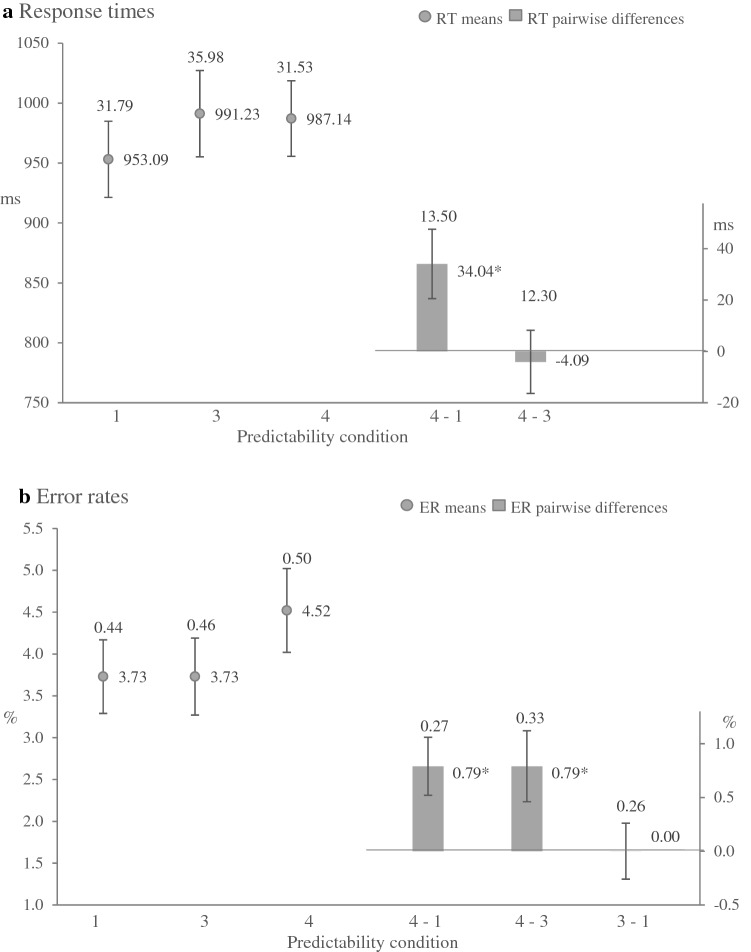
Results of Experiment 2. **a** Mean RTs for each condition (dots; 1 = expected colour and task, 3 = neutral colour and expected task, 4 = neutral colour and unexpected task) and corresponding pairwise differences (bars). **b** Mean ERs for each condition (dots) and corresponding pairwise differences (bars). Numbers beside the dots and the bars indicates the numerical values of means. Error bars represents ± 1 SEM, numbers above error bars are the numerical values of the SEMs. *p < .05. The results are averaged across FPs and task transition

We screened and processed the data in the same way as in Experiment 1 and conducted three-factor repeated measures ANOVAs, with the factors for the foreperiod (500 vs. 1500 ms), transition (repetition vs. switch) and predictability condition (which included different types of trials) separately for RTs and ERs. Figure 3 shows the mean RTs and the mean ERs of each predictability condition and the differences between the conditions. In the Appendix, we have provided an overview of the results of ANOVAs in Tables 4, 5 and 6.

### Expected colour and task vs. neutral colour and unexpected task

**Response Times** Responses were faster after the short foreperiod than after the long foreperiod, *F* (1, 63) = 15.88, *p* < .01, η_p_^2^ = .2. Responses in trials after task repetitions were faster than in trials after task switches, *F* (1, 63) = 65.71, *p* < .01, η_p_^2^ = .51. Furthermore, the pronounced main effect of predictability condition indicated that participants clearly formed time-based expectancy, *F* (1, 63) = 6.36, *p* = .01, η_p_^2^ = .09, as they responded on average 34 ms faster in trials with expected colour and task than in trials with neutral colour and unexpected task (pairwise differences 3 - 1, Fig. 3a). A corresponding Bayesian ANOVA provided no support for the null model (no difference between the predictability conditions where the factors foreperiod and transitions were included), *BF*_*01*_ = .59.

**Error Rates** Participants produced significantly fewer errors in trials with task repetitions than in trials with task switches *F* (1, 63) = 5.39, *p* = .02, η_p_^2^ = .08. The main effect of predictability condition, *F* (1, 63) = 8.25, *p* = .01, η_p_^2^ = .12, also attained significance. As illustrated in the Fig. 3b (pairwise differences 3 - 1), the error rate was lower in trials with expected colour and task than in trials with neutral colour and unexpected task. Neither the main effect of foreperiod nor any interactions were significant. A corresponding Bayesian ANOVA provided no evidence for the null model, *BF*_*01*_ = .89.

### Neutral colour and expected task vs. neutral colour and unexpected task

**Response Times** The main effects of foreperiod, *F* (1, 63) = 16.45, *p* < .01, η_p_^2^ = .21 and transition, *F* (1, 63) = 42.63, *p* < .01, η_p_^2^ = .4, were again significant. There was no significant difference between trials with expected task and trials with unexpected task, *F* (1, 63) = .11, *p* = .74, η_p_^2^ = .00 (pairwise differences 3 - 2, Fig. 3a), but the predictability condition interacted with task transition *F* (1, 63) = 5.14, *p* = .03, η_p_^2^ = .08, such that switch costs were higher in trials with expected task, *M* = 154 ms, than in trials with unexpected task, *M* = 98. The Bayesian repeated measures ANOVA revealed strong evidence for the absence of a time-based expectancy effect, *BF*_*01*_ = 1.46.

**Error Rates** Once more, we observed a significant main effect of task transition, *F* (1, 63) = 4.48, *p* = .04, η_p_^2^ = .07, as well as a significant main effect of predictability condition, *F* (1, 63) = 5.72, *p* = .02, η_p_^2^ = .08, with a lower ER in trials with expected task in comparison to trials with unexpected task. The Bayesian analysis did not support the null model without the main effect of predictability condition, *BF*_*01*_ = 2.04.

### Expected colour and task vs. neutral colour and expected task

The ER analyses revealed an effect of time-based expectancy in trials with expected colour and task as well as in trials with neutral colour and expected task. Therefore, we analysed whether the ERs in these two conditions differed.

Participants produced significantly fewer errors in trials with task repetitions than in trials with task switches, *F* (1, 63) = 3.63, *p* = .06, η_p_^2^ = .06. Neither the main effect of foreperiod nor the main effect of predictability condition or any interactions reached significance (pairwise differences 2 - 1, Fig. 3b). The Bayesian ANOVA revealed a strong evidence for the absence of difference between ERs in trials with expected colour and task and ERs in trials neutral colour and expected task, *BF*_*01*_ = 11.09.

### Cross-experimental analysis

In contrast to Experiment 1, we clearly observed the time-based expectancy effect in Experiment 2 in both RT and ER analyses. To examine whether the strengthened predictability yielded this effect, we conducted a cross-experiment analysis on RT and ER data. A mixed-design ANOVA contained within-subjects factors foreperiod (short vs. long), transition (repetition vs. switch), predictability (expected colour and task vs. neutral colour and unexpected task) and the between-subjects factor the experiments (1 vs. 2).

**Response times** Three significant main effects of foreperiod, *F* (1, 125) = 29.3, *p* < .01, η_p_^2^ = .19, transition, *F* (1, 125) = 102.3, *p* < .01, η_p_^2^ = .45 as well as predictability condition, *F* (1, 125) = 12.06, *p* = .00, η_p_^2^ = .08, were observed. The main effect of the between factor experiment was not significant, *F* (1, 125) = .43, *p* = .51, η_p_^2^ < .01 nor did it interact with any of the other three main effects (see Table 6 in Appendix). An analogous Bayesian repeated measures ANOVA showed that the null model for the interaction between the predictability condition and the experiment (no difference in time-based colour expectancy between the two experiments) was 4 times as likely as the alternative model, *BF*_01_ = 4.46.

**Error rates** Two main effects were significant, but the experiment did not interact with any of the main effects. ERs were lower for task repetitions than for task switches, *F* (1, 125) = 8.02, *p* < .001, η_p_^2^ = .06 and this effect did not differ between experiments, *F* (1, 125) = .09, *p* = .76, η_p_^2^ = .0. The main effect of predictability condition was also significant, *F* (1, 125) = 1.57, *p* < .001, η_p_^2^ = .08 and again this effect did not differ between experiments, *F* (1, 125) = .14, *p* = .71, η_p_^2^ = .0. Neither the main effects of the foreperiod, the experiment, nor any other interactions gained significance. The Bayesian repeated measures ANOVA revealed strong evidence for the absence of difference in time-based colour expectancy between experiments, *BF*_*01*_ = 19.45.

### Exploratory analysis

**Fig. 4:**
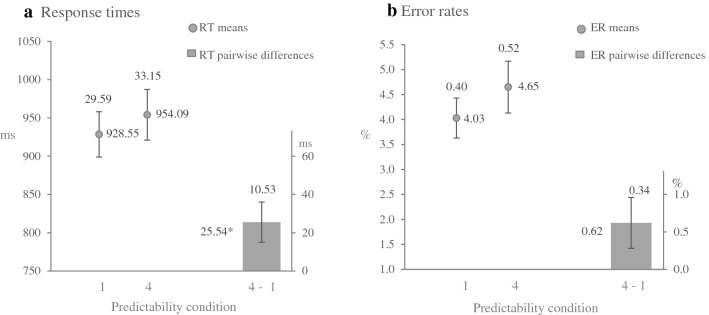
Results of exploratory analysis in Experiment 1. **a** Mean RTs for each condition (dots; 1 = expected colour and task, 4 = neutral colour and unexpected task) and corresponding pairwise differences (bars). **b** Mean ERs for conditions 1 and 4 (dots) and corresponding pairwise differences (bars). Numbers beside the dots and the bars indicate the numerical values of means. Error bars represent ± 1 SEM, numbers above error bars are the numerical values of the SEMs. *p < .05. The results are averaged across FPs and task transition

Surprisingly, although the effect on time-based expectancy was only significant in Experiment 2, the cross-experiment comparison revealed no significant difference in the time-based expectancy effect between the two experiments. The reason might be due to the different predictability condition that was included in the cross-experimental analysis. In Experiment 1, we compared the first condition with frequent combinations of foreperiod to colour and task (expected colour and task) as opposed to the second condition with infrequent combinations of foreperiod to colour and task (unexpected colour and task). In the cross-experiment analysis, we used trials with neutral colour and unexpected task instead. Thus, we conducted a further analysis of Experiment 1 comparing trials with expected colour and task (first condition) against trials with neutral colour and unexpected task (fourth condition). Figure 4 illustrates the differences in RTs and ERs between the predictability conditions and Table 7 in Appendix gives a total overview of the results.

**Response times** As expected, the main effect of foreperiod, *F* (1, 62) = 13.57, *p* < .001, η_p_^2^ = .18 and the main effect of transition, *F* (1, 62) = 39.09, *p* < .001, η_p_^2^ = .39, were significant. More importantly, the main effect of predictability condition also gained significance, *F* (1, 62) = 5.88, *p* = .02, η_p_^2^ = .09. The corresponding Bayesian analysis revealed no evidence for the null model (no difference in predictability conditions), *BF*_*01*_ = 1.4.

**Error rates** Neither the main effects nor the interactions were significant. An analogous Bayesian repeated measures ANOVA showed only weak evidence for the absence of the main effect of predictability condition, *BF*_*01*_ = 2.58.

**Post-experimental questionnaire** According to the post-experimental questionnaire, none of the participants noticed any temporal regularities in the experiment.

## Discussion

As in Experiment 1, participants showed typical task switching costs and faster responses in trials with short foreperiods compared to trials with long foreperiods. More importantly, the large and significant main effect of predictability observed in Experiment 2 indicated that the participants successfully formed time-based expectancy. Moreover, the analysis of RTs showed that performance improved, when the colour and the task occurred frequently after the specific foreperiod (expected colour and task), but not when only the task was frequent assigned to the specific foreperiod (neutral colour), favouring a perceptual preparation as an explanation for the time-based expectancy in the current study. On the other hand, participants produced more correct responses in trials with expected colour and task as well as in trials with neutral colour and expected task compared to trials with neutral colour and unexpected task. Additionally, no significant difference was observed between error rates in both types of trials with expected task, i.e. expected colour vs. neutral colour. These observations reference to a time-based expectancy for task.

At first glance, the main analysis in Experiment 2 supports our interpretation that the absence of a time-based expectancy effect in Experiment 1 was due to the induced probability being too weak. Instead, and in line with the study by Aufschnaiter et al., (2018a, 2018b), the additional cross-experiment analysis indicated that the effect of time-based colour expectancy did not differ in the two experiments. Given this unexpected finding, we further analysed the data from Experiment 1 by comparing trials with expected colour and task against trials with neutral colour and expected task. Please note that in the initial analysis in Experiment 1, we used trials with unexpected colour and task, i.e. colour and task appeared particularly infrequently after a specific foreperiod. The results of the RTs analysis revealed evidence for time-based expectancy of colour. Thus, the two experiments overall provided evidence for time-based expectancy for a task indicator from RTs and a tentative hint for time-based expectancy for task from ERs.

## General discussion

In the two experiments, we tested whether time-based expectancy in task switching studies is due to perceptual colour expectancy or to task-set expectancy. We formulated three not fully mutually exclusive hypotheses. Firstly, we predicted that if the effect of time-based expectancy were exclusively due to task expectancy, we should observe improved performance in two conditions with expected task, that is to say with expected and with neutral colours and there should be no performance difference between these two conditions. Secondly, if the effect of time-based colour and task expectancies were additive, we would expect improved performance in the two conditions with expected task, with the time-based expectancy effect being stronger for the expected colours than for neutral colours. As a third option, we assumed that if the time-based expectancy effect occurred only in conditions with expected colour and the task, then the time-based expectancy should only facilitate perceptual colour processing.

To discriminate between these three options, we conducted two experiments. The results of both experiments together provide strong support for time-based expectancy of a task indicator, i.e. colour. We observed significantly faster RTs in trials with expected colour and task compared to trials with neutral colour and unexpected task. This finding was further supported by a Bayesian analysis that revealed evidence in favour of the time-based expectancy effect of colour in Experiment 2. Our results demonstrated that a time-based expectancy effect is not task-related, because when comparing two conditions with neutral colour, we did not observe performance differences between expected and unexpected tasks.

Yet, there are also indications in the data suggesting that the second hypothesis should not be prematurely rejected and that time-based expectancy for tasks contribute to the effect. In the second experiment, participants performed more accurately in trials with neutral colour and expected task than in trials with neutral colour and unexpected task. At the same time, no difference in accuracy was found between trials with expected colour and task and trials with neutral colour and expected task. However, this is the only evidence that time-based task expectancy contributes to the effect. Therefore, based on the present findings, we favour the perceptual explanation for the time-based expectancy effect in a task switching scenario.

Please consider, when interpreting the results from a learning perspective, the time-based expectancy refers to the implicit learning of foreperiod-event contingencies. We assume that participants build strong associations between frequent combinations of foreperiod to event without being aware of the different frequencies. In line with previous studies (Aufschnaiter et al., 2018a, 2018b), participants failed to notice any temporal regularities in both experiments, providing clear evidence for the implicit character of time-based expectancy.

It is important to note that our conclusions may be limited by the fact that in the present experiments the predictability of colours (90% in Experiment 1, 100% in Experiment 2) was always stronger than the predictability of task-set (70% in Experiment 1, 75% in Experiment 2). In comparison, in the study by Aufschnaiter et al., (2018a, 2018b), the task indicator (i.e. the stimulus colour) was predictable to the same degree as the task itself. Additionally, participants had to manage the higher requirements contained in the present design; firstly they had to be aware of four colours and, secondly, had to assign two colours to each task. Beyond that, only one of the two colours which indicated each task was expected while the other one was neutral. This circumstance may have led participants to use the more efficient strategy of building time-based expectancy for the task component, which was more frequently associated with the specific foreperiod, in this case the coloured task indicator and to neglect the task-set as a less frequent task component. In order to differentiate whether the different degrees of predictability for the colour and for the task-set in the present experiment in fact influenced the direction of time-based expectancy, future experiments should include conditions where task indicators do not more greatly benefit from the temporal predictability than the task itself.

At first sight, the more complex experimental setting in the current study and the lower degree of predictability of 90% (for colour) are the reasons why significant time-based expectancy effect of colour was initially not observed in Experiment 1. Indeed, strengthening the predictability of colour to 100% in Experiment 2 allowed the provision of clear evidence for time-based colour expectancy. Hence, it seems that by performing more difficult tasks, participants can build time-based expectancy only if the foreperiod predicts the upcoming event (e.g. colour) with 100% validity. However, as similar predictability conditions (expected colour and task vs. neutral colour and unexpected task) were included in the cross-experiment analysis, a significant time-based expectancy effect of colour was found and this effect did not differ between experiments. Moreover, an advanced analysis of the predictability conditions with expected colour and task as well as with neutral colour and unexpected task in Experiment 1 also provided evidence for time-based colour expectancy. Therefore, we could not conclude that the complexity of the task played a critical role in the observation of a time-based expectancy in our study. Further research is required to investigate whether an even smaller degree of predictability than in the current design would impair the strength of the time-based expectancy effect.

For theoretical models of time-based expectancy, the present findings suggest that time-based expectancy facilitates basic cognitive functions such as motor processes (Thomaschke & Dreisbach, 2013; Thomaschke et al., 2016) as well as perceptual processes (Rieth & Huber, 2013; Thomaschke et al., 2016) instead of complex cognitive control processes. This conclusion is supported by a lack of interaction between transition and time-based expectancy. Since cognitive processes involved in task switch and task repetition trials differ (see Introduction), an expectancy for switch-related processes should have influenced task switching and task repetition differently. In line with most previous time-based expectancy studies which apply task switching scenarios (Aufschnaiter et al., 2018a, 2018b, except Experiment 1; 2020; 2021), such an effect could not be detected in our study. However, this interpretation conflicts with a study by Wendt and Kiesel (2012), which demonstrated a benefit from time-based expectancy for conflict adaptation. The authors investigated whether the time-based expectancy of conflict in flanker tasks can improve attentional adjustment and showed that conflict adaptation was modulated by contingencies involving combinations of time and conflict proportions and that learning these combinations influenced the flanker interference. Therefore, if higher cognitive functions benefit from time-based expectancy, we cannot rule out that the task-set processing in task switching scenarios can be facilitated, if the task is predictable by the foreperiod to the same amount as the task indicator.

Previous evidence of task-specific temporal preparation was observed in the variable foreperiod paradigm (e.g. Niemi, & Näätänen, 1981) showing faster RTs for the long rather than the short foreperiod and, more importantly, this variable foreperiod effect was larger for predictable tasks than for unpredictable tasks (Schröter et al., 2015). This is partly in line with the results of the present study, which showed temporal preparation for visual processing of a specific task indicator. However, Schröter et al. (2015) induced task expectancy by asking participants to switch tasks in each trial. That is, in blocks with an alternation between two tasks the participants always knew *which task* to expect in current trial, but it was uncertain *when* the expected event would occur, as the foreperiods were randomly intermixed within the blocks. In contrast, the time-based expectancy is frequency-induced. Here, the duration of foreperiod itself is critical for the effect of temporal preparation so that a certain event, i.e. *which task*, can be expected in a certain point of time, i.e. *when* (Thomaschke & Dreisbach, 2015). Thus, there are presumably different mechanisms of temporal preparation in the variable foreperiod paradigm and in the time-event correlation paradigm, which is the basis for time-based expectancy. However, despite the differences, both studies revealed clear evidence that the temporal preparation is not only general but also event-specific.

In the present study, RTs were faster after the short foreperiod than after the long foreperiod and identical patterns were also reported in task switching studies from Aufschnaiter et al., (2018a, 2018b). These findings are opposite to the variable foreperiod effect (Niemi, & Näätänen, 1981; Schröter et al., 2015). However, both opposing observations can be explained by the theory of strategic adaptation of response readiness (Niemi, & Näätänen, 1981). According to the theory, the readiness to respond to an upcoming event is highest directly before the anticipated onset of the imperative stimulus. In the variable foreperiod paradigm, the probability of an event appearance increases with the age of a foreperiod (Näätänen, 1970), leading to a higher response readiness and thus generating performance benefit at the long foreperiod. On the other hand, time-based event expectancy and consequently the readiness to process the respective event is activated shortly before the time point at which the event frequently occurred in the past (Thomaschke & Dreisbach, 2015). Thus, it might be that each time, shortly before the passage of the short foreperiod, the readiness to process the respective event is at its highest and decreases if the short foreperiod passes without the occurrence of the event. Furthermore, the readiness to process the event associated with the short foreperiod might not vanish completely at the end of the long foreperiod and might somehow interfere with the readiness to process the event which is related with the long foreperiod. If this assumption is true, the interference of two activated preparatory states would lead to a performance disadvantage for the long foreperiod. However, this speculative explanation is limited by the observation that the effect of foreperiod did not interact with the effect of time-based expectancy in the present study. In addition, a tendency towards faster responses after a short time interval than after a long time interval had been previously observed in previous task switching studies without time-based expectancy that used randomly varied intervals within a block of trials (Gade & Koch, 2005; Rogers & Monsell, 1995).

With regard to task switching theories, our findings suggest that time-based expectancy might not be a new source of task preparation. It seems that under the increased requirements of multitasking, our cognitive system shows a flexible adaptation to the existing circumstances and establishes resource-saving operations, such as time-based expectancy for the most common event. Although time-based expectancy is important in task switching, it supports performance by speeding up task-indicator perception rather than by facilitating task-set implementation.

Taken together, our results are consistent with previous studies that already demonstrated that time-based expectancy speeded up visual processing (Rieth & Huber, 2013; Thomaschke et al., 2016). Moreover, the current results provide evidence that this mechanism not only occurs when executing a simple single task, but that time-based expectancy also improves general task switching performance, that is, both task repetition and task switch, by accelerating the visual processing of the task indicator.

The finding is relevant to the design of human-computer interfaces, particularly in scheduling system delays. In the interaction with computers, system delays refer to the waiting time between user input and a program’s response. This waiting time is caused by several, usually simultaneously running computing processes. For example, during the download and installation of new smartphone apps, the computer system has to manage in parallel the download and the installation procedures as well as the dialogue with the user which provides the information about the forthcoming steps. In modern computer systems, the different processes or the different system users often share one single processor. The resulting scheduling of the processes leads to system delays of different length (Blazewicz et al., 2007). Several previous findings showed that such variable delays reduce user satisfaction and performance (Fischer et al., 2005; Weber et al., 2013). On the contrary, a study conducted by Thomaschke and Haering (2014) showed that performance improved if delays were variable but highly predictive for the upcoming event. Looking at the results of the study by Aufschnaiter et al. (2018a), it could be argued that multitasking interfaces should be designed in such a way that the variable system delays predict different tasks in order to make the entire human-computer interaction more efficient by reducing the user’s RT. Critically, however, our present results indicate that it is essential that the delays predict task instruction. A delay that only predicts the task (e.g. answering phone calls) would not improve user performance unless the different task indicators (pop-up messages, phone ringing, light signal) are also predictable by time.

Proceeding from previous findings that temporal predictability not only improves the execution of a single task but also enhances performance when switching between tasks, the present study systematically examined the influence of time-based expectancy on different cognitive processes involved in task switching. Overall, our results suggest that the time-based expectancy can facilitate visual processing and hence improve performance in the task switching scenario. This finding is not only important for the understanding of basic cognitive mechanisms but also has practical implications for the design of human-machine interfaces.

## Appendix

See Tables 2, 3, 4, 5, 6 and 7.

**Table 2:** Results of RTs ANOVAs in Experiment 1

	Condition 1 vs. 2			Condition 3 vs. 4		
Factors	*F*	*p*	η_p_^2^	*F*	*p*	η_p_^2^
Foreperiod (FP)	8.59	0.00	0.12	17.35	0.00	0.22
Transition (Tr)	28.44	0.00	0.31	36.03	0.00	0.37
Predictability condition (PrC)	3.65	0.06	0.06	0.27	0.61	0.00
FP × Tr	0.15	0.70	0.00	0.21	0.65	0.00
FP × PrC	0.12	0.73	0.00	0.10	0.76	0.00
Tr × PrC	0.31	0.58	0.00	0.41	0.53	0.01
FP × Tr × PrC	1.02	0.32	0.02	1.73	0.19	0.03

**Table 3:** Results of ERs ANOVAs in Experiment 1

	Condition 1 vs. 2	Condition 3 vs. 4				
Factors	*F*	*p*	η_p_^2^	*F*	*p*	η_p_^2^
Foreperiod (FP)	0.05	0.50	0.01	0.06	0.8	0.00
Transition (Tr)	4.52	0.04	0.07	4.80	0.03	0.07
Predictability condition (PrC)	0.11	0.75	0.00	2.74	0.1	0.04
FP × Tr	0.26	0.61	0.00	5.82	0.02	0.09
FP × PrC	0.02	0.9	0.00	1.57	0.21	0.03
Tr × PrC	0.28	0.6	0.01	3.07	0.08	0.05
FP × Tr × PrC	0.01	0.92	0.00	0.16	0.69	0.00

**Table 4:** Results of RTs ANOVAs in Experiment 2

	Condition 1 vs. 3			Condition 2 vs. 3			Condition 1 vs. 2		
Factors	*F*	*p*	η_p_^2^	*F*	*p*	η_p_^2^	*F*	*p*	η_p_^2^
Foreperiod (FP)	15.88	0.00	0.20	16.45	0.00	0.21	1.08	0.30	0.02
Transition (Tr)	65.77	0.00	0.51	42.63	0.00	0.4	44.65	0.00	0.42
Predictability condition (PrC)	6.36	0.01	0.09	0.11	0.71	0.00	13.53	0.00	0.18
FP × Tr	0.04	0.85	0.00	0.31	0.58	0.01	3.21	0.08	0.05
FP × PrC	2.05	0.16	0.03	1.09	0.3	0.02	0.21	0.65	0.00
Tr × PrC	1.71	0.20	0.03	5.14	0.03	0.08	2.40	0.13	0.04
FP × Tr × PrC	2.06	0.16	0.03	3.39	0.07	0.05	0.47	0.5	0.01

**Table 5:** Results of ERs ANOVAs in Experiment 2

	Condition 1 vs. 3			Condition 2 vs. 3			Condition 1 vs. 2		
Factors	*F*	*p*	η_p_^2^	*F*	*p*	η_p_^2^	*F*	*p*	η_p_^2^
Foreperiod (FP)	0.01	0.92	0.00	0.41	0.52	0.01	1.21	0.28	0.02
Transition (Tr)	5.39	0.02	0.08	4.48	0.04	0.07	3.36	0.06	0.06
Predictability condition (PrC)	8.25	0.01	0.12	5.72	0.02	0.08	0.00	0.98	0.00
FP × Tr	0.45	0.51	0.01	0.69	0.41	0.01	0.44	0.51	0.01
FP × PrC	0.44	0.51	0.01	0.79	0.38	0.01	0.46	0.5	0.01
Tr × PrC	0.11	0.75	0.00	0.15	0.7	0.00	0.06	0.82	0.00
FP × Tr × PrC	0.13	0.72	0.00	1.56	0.22	0.02	3.56	0.06	0.05

**Table 6:** Results of cross-experiment ANOVAs for RTs and ERs

	RTs			ERs		
Factors	*F*	*P*	η_p_^2^	*F*	*p*	η_p_^2^
Experiment (Ex)	0.43	0.51	0.00	0.12	0.73	0.00
Foreperiod (FP)	29.30	0.00	0.19	0.23	0.64	0.00
Transition (Tr)	102.31	0.00	0.45	8.02	0.01	0.06
Predictability condition (PrC)	12.06	0.00	0.09	10.57	0.00	0.08
Ex × FP	0.00	0.99	0.00	0.4	0.53	0.00
Ex × Tr	1.04	0.31	0.01	0.09	0.77	0.00
Ex × PrC	0.25	0.62	0.00	0.14	0.71	0.00
FP × Tr	0.01	0.91	0.00	0.03	0.87	0.00
FP × PrC	0.73	0.39	0.01	1.26	0.26	0.01
Tr × PrC	6.73	0.01	0.05	0.18	0.68	0.00
Ex × FP × Tr	0.16	0.69	0.00	1.33	0.25	0.01
Ex × FP × PrC	1.47	0.23	0.01	0.03	0.85	0.00
Ex × Tr × PrC	0.65	0.42	0.00	0.7	0.41	0.01
FP × Tr × PrC	2.83	0.09	0.02	3.2	0.08	0.03
Ex × FP × Tr × PrC	0.56	0.46	0.00	1.8	0.18	0.01

**Table 7:** Results of RTs and ERs ANOVAs with Predictability Conditions 1 and 4 in Experiment 1

	RTs			ERs		
Factors	*F*	*p*	η_p_^2^	*F*	*p*	η_p_^2^
Foreperiod (FP)	13.56	0.00	0.18	0.77	0.38	0.01
Transition (Tr)	39.09	0.00	0.39	2.94	0.09	0.05
Predictability condition (PrC)	5.88	0.02	0.09	3.41	0.07	0.05
FP × Tr	0.15	0.70	0.00	0.97	0.33	0.02
FP × PrC	0.07	0.79	0.00	0.85	0.36	0.01
Tr × PrC	5.42	0.02	0.08	0.67	0.42	0.01
FP × Tr × PrC	0.79	0.38	0.01	3.88	0.05	0.06

## Footnotes

1 Please note, the effect size η_p_^2^ = .28 in Experiment 3 (70% predictability) was even descriptively larger.

## Declarations

### Public significance statement

Understanding the cognitive strategies that help us cope with multitasking situations is of great importance in our daily lives. One of such strategies is time-based task expectancy in task switching, which is the observation that a fixed waiting time used for a certain task becomes predictive for that type of task. In two experiments, we found that this time-based expectancy enables humans to faster recognize visual stimuli that signal the task type. This finding has also practical implications for the design of human-machine interfaces.
